# Donor Transmission of Melanoma Following Renal Transplant

**DOI:** 10.1155/2012/764019

**Published:** 2012-11-04

**Authors:** Kathryn T. Chen, Anthony Olszanski, Jeffrey M. Farma

**Affiliations:** ^1^Department of Surgical Oncology, Fox Chase Cancer Center, Philadelphia, PA 19147, USA; ^2^Department of Medical Oncology, Fox Chase Cancer Center, Philadelphia, PA 19147, USA

## Abstract

Donor transmission of melanoma is one of the more common and lethal of recipient malignancies, often presenting with systemic disease. Although some patients may receive durable remission of melanoma following explantation of the allograft and withdrawal of immunosuppression, donor transmission of melanoma is fatal in most patients. Here we present a case of a 44-year-old male who developed metastatic melanoma following renal transplant.

## 1. Case Report

**Figure 1:**
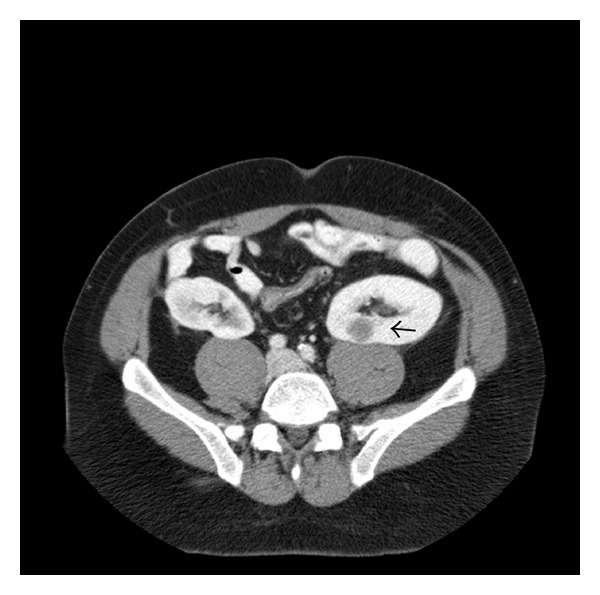
Metastatic melanoma, left kidney (arrow).

A 44-year-old African American male with end-stage renal disease secondary to focal sclerosing glomerulonephritis underwent sequential renal transplants. He received his first cadaveric renal transplant in 2008, and when that failed, he underwent a second cadaveric renal transplant two years later. Subsequent to transplantation, the recipient of the hepatic allograft developed cerebral melanoma metastases. The donor was not known to have a history of melanoma prior to death. Our patient was notified and further work-up was pursued. He underwent CT of the chest, abdomen, and pelvis, as well as a brain MRI. This demonstrated three indeterminate lesions on the left transplant kidney (Figure 1), which were subsequently biopsied and demonstrated to be metastatic melanoma. No other foci of disease were demonstrated.

At this point, the patient underwent transplant nephrectomies of both kidneys; these took place approximately one year following his original transplant. Final pathology on the left kidney demonstrated 2 foci of metastatic melanoma; one focus was 1.5 cm, and the other was 1.8 cm. Vascular invasion and penetration of the renal capsule were both present. Immunohistochemical staining was performed with S100, HMB45, and MELAN-A, which were all positive. Immunosuppressive medications were tapered and discontinued.

Four months later, the patient was referred to medical oncology for further follow up. On physical exam, he was found to have a 1 cm firm subcutaneous nodule of the left anterior thigh, concerning for a metastatic deposit. Review of his prior CT scans revealed that imaging did not include this area. Wide local excision of the thigh lesion yielded a 1-cm nodule; pathology was consistent with necrotic melanoma. The lesion was sent for BRAF genomic analysis, which was negative for mutation. A PET/CT and brain MRI were also ordered.

The PET scan revealed multiple foci of uptake on skin surfaces, bone, and in the subcutaneous tissues, in the extremities and abdominal wall. A left temporal lobe lesion was also noted; MRI corroborated the impression of a metastatic 3 × 2 cm lesion with corresponding vasogenic edema and mass effect. Following these scans, the patient developed expressive aphasia with word finding difficulties and headaches. Steroids were instituted and emergent neurosurgical evaluation was sought. The patient underwent resection of the left temporal lobe lesion. Pathology was consistent with metastatic melanoma. Postoperatively, he underwent whole-brain radiation with a total of 37.5 Gray in 15 fractions. He regained all of his baseline cognitive ability. 

After the completion of whole brain radiation, the patient received a total of four cycles of ipilimumab (3 mg/kg) which he tolerated well. He developed a mild pruritus on treatment, controlled with topical creams. His posttreatment PET-CT scans demonstrated a complete response with no residual metabolically active lesions.

The patient is currently 16 months out from his donor nephrectomies, and without evidence of disease. He will continue to undergo surveillance by physical exam and PET-CT every 3-4 months for the next year, and then biannually following this.

## 2. Review of the Literature and Discussion

Although we are cautiously optimistic for a durable remission in our patient, many patients who receive donor transmission of melanoma are not so fortunate. As with our patient, donor transmission of melanoma generally presents in a metastatic fashion, sometimes with cutaneous manifestation or to other solid organs, but other times with miliary-like mucosal or peritoneal lesions [1, 2]. Recipients often die of complications related to uncontrolled metastatic disease [1, 3, 4].

Transmission of donor related malignancy is extremely rare. A study from the Organ Procurement and Transplantation Network/United Network for Organ Sharing reported 21 donor related malignancies out of 34,933 cadaveric donors over a 7-year period [5]. However, melanoma is noted to have one of the highest transmission rates at 74%, and a reported subsequent mortality of 58% [6]. The manifestation of metastatic disease occurs commonly within the first year [1, 3]. Interestingly, although the majority of donors do not have a reported history of melanoma, the cause of death in donors is often attributed to intracranial hemorrhage, suggesting potentially undiagnosed metastatic disease [7]. In two case reports, previous donor history of melanoma was known, but treatment and presumed cure occurred 16 and 32 years prior to donor death and subsequent transmission of melanoma to recipients [2, 8]. This protracted latency period demonstrates the ability of melanoma tumor cells to lay dormant, and thus, any history of melanoma, no matter how remote, is generally considered an absolute contraindication to transplant [6, 8]. However, this is not the worldwide consensus, and there is less opposition towards donors with a history of in-situ melanoma, although there is a lack of data with regards to this [9].

The mechanism of transmission of melanoma is not entirely clear, but presumably is a result of either circulating tumor cells in the vasculature or quiescent cells that remain in the parenchyma of transplanted organs. It is known that patients with advanced stage melanoma may have detectable levels of circulating tumor cells on the level of 1 tumor cell per 10^6^ normal red blood cells [10, 11]. Although it is unclear what the significance of circulating tumor cells is, there appears to be some correlation with stage and survival [10, 12]; thus, it seems unlikely that donors who have a remote history of melanoma continue to harbor active circulating tumor cells. On the other hand, there are case reports of donor-disseminated melanoma in which the renal and liver allografts developed tumors, but not the recipient of the cardiac allograft [1, 13]. Furthermore, some case reports note disease confined to the allograft, suggestive of dormant melanoma cells within the parenchyma that become active with immunosuppression [13, 14].

Although donor-transmitted melanoma is highly lethal, there is definitive evidence that cure, even in the face of initial metastatic disease, is achievable with explantation of the affected allograft and withdrawal of immunosuppression. Certainly the circumvention of melanoma requires an immunocompetent host; an early study of 14 patients who developed de novo cutaneous melanoma following renal transplant demonstrated lack of cellular response to tumor in the excised specimen [15]. Furthermore, the development of de novo cutaneous melanoma is accelerated in transplant patients in general population studies [16, 17]. At any rate, durable remission of metastatic melanoma has been reported in several case reports with transplant nephrectomy, withdrawal of immunosuppression, and in some cases, institution of immunotherapy [8, 13, 18, 19]. In one of these, the recipient was able to be retransplanted three years following explant, without evidence of melanoma at follow-up [13].

Upon recognition of possible transplant-related melanoma transmission, our patient was advised to undergo nephrectomy, which he initially resisted. However, he underwent the recommended nephrectomy after a CT scan revealed renal lesions. Subsequently, he was weaned from all immunosuppressant medications. The use of erythropoietin was also discontinued. Despite these measures, upon first assessment with oncology, he was found to have metastatic disease. After appropriate treatment of his brain lesions, he received the anti-CTLA4 inhibitor, ipilimumab, at a dose of 3 mg/kg. As monoclonal antibodies are felt to undergo receptor-mediated disposition, no dose modification was made; he continued on hemodialysis throughout his treatment course and tolerated treatment well. His first post-ipilimumab PET/CT demonstrated a complete response, which has been maintained to date. 

Ipilimumab's mechanism-of-action is felt to be related to inhibition of the negative T-cell regulatory receptor, CTLA-4 [20]. Appropriate activation of T-cells requires, in part, appropriate antigen expression by an antigen-presenting cell. The use of ipilimumab, therefore, may lead to nonspecific T-cell activation and this can result in autoimmune-like toxicities, which have been well described. In our patient, the melanoma was acquired via cadaveric transplant. It is postulated that this resulted in a more specific APC/T-cell interaction in our patient, and perhaps was critical in the ensuing development of a complete response. In summary, the use of ipilimumab in this hemodialysis-dependent individual with a cadaveric-related melanoma was well tolerated without dose modification and has led to a durable complete response to date.
